# Effect of the normalized prescription isodose line on the magnitude of Monte Carlo vs. pencil beam target dose differences for lung stereotactic body radiotherapy

**DOI:** 10.1120/jacmp.v17i4.5965

**Published:** 2016-07-08

**Authors:** Dandan Zheng, Qinghui Zhang, Xiaoying Liang, Xiaofeng Zhu, Vivek Verma, Shuo Wang, Sumin Zhou

**Affiliations:** ^1^ Department of Radiation Oncology University of Nebraska Medical Center Omaha NE USA; ^2^ Department of Radiation Medicine Northwell Health New York NY USA; ^3^ University of Florida Health Proton Therapy Institute Jacksonville FL USA

**Keywords:** lung SBRT, prescription isodose line, Monte Carlo, pencil beam

## Abstract

In lung stereotactic body radiotherapy (SBRT) cases, the pencil beam (PB) dose calculation algorithm is known to overestimate target dose as compared to the more accurate Monte Carlo (MC) algorithm. We investigated whether changing the normalized prescription isodose line affected the magnitude of MC vs. PB target dose differences. Forty‐eight patient plans and twenty virtual‐tumor phantom plans were studied. For patient plans, four alternative plans prescribed to 60%, 70%, 80%, and 90% isodose lines were each created for 12 patients who previously received lung SBRT treatments. Using 6 MV dynamic conformal arcs, the plans were individually optimized to achieve similar dose coverage and conformity for all plans of the same patient, albeit at the different prescription levels. These plans, having used a PB algorithm, were all recalculated with MC to compare the target dose differences. The relative MC vs. PB target dose variations were investigated by comparing PTV D95, Dmean, and D5 loss at the four prescription levels. The MC‐to‐PB ratio of the plan heterogeneity index (HI) was also evaluated and compared among different isodose levels. To definitively demonstrate the cause of the isodose line dependence, a simulated phantom study was conducted using simple, spherical virtual tumors planned with uniform block margins. The tumor size and beam energy were also altered in the phantom study to investigate the interplay between these confounding factors and the isodose line effect. The magnitude of the target dose overestimation by PB was greater for higher prescription isodose levels. The MC vs. PB reduction in the target dose coverage indices, D95 and V100 of PTV, were found to monotonically increase with increasing isodose lines from 60% to 90%, resulting in more pronounced target dose coverage deficiency at higher isodose prescription levels. No isodose level‐dependent trend was observed for the dose errors in the target mean or high dose indices, Dmean or D5. The phantom study demonstrated that the observed isodose level dependence was caused by different beam margins used for the different isodose levels: a higher prescription line required a larger beam margin, leading to more low‐density lung tissues in the field and, therefore, larger dose errors at the target periphery (when calculated with PB). The phantom study also found that the observed isodose level dependence was greater for smaller targets and for higher beam energies. We hereby characterized the effect of normalized prescription isodose line on magnitude of PTV dose coverage as calculated by MC vs. PB. When comparing reported MC dose deficiency values for different patients, the selection of prescription isodose line should be considered in addition to other factors known to affect differences in calculated doses between various algorithms.

PACS number(s): 87.55.kh, 87.55.dk, 87.55.de

## I. INTRODUCTION

A key issue facing the fidelity and efficacy of lung stereotactic body radiotherapy (SBRT) is the accuracy of dose calculation. These technical challenges arise in part from the heterogeneous interface between the higher‐density tumor and the lower‐density lung tissue, which can be accentuated by the hypofractionated nature of SBRT. Partially as a result of these uncertainties, it is known that the Monte Carlo (MC) dose calculation algorithm more accurately models the dose distribution as compared to the pencil beam (PB) algorithm.^(1)^ However, clinical implementation of MC‐based lung SBRT planning has been limited due to various challenges, despite the rapid advancement of fast MC algorithms in treatment planning systems. One such challenge is the vast variations in dose differences between doses calculated by MC and other algorithms such as PB. For example, in a series of 53 patients, reduction of the planning target volume (PTV) D95 by the MC algorithm, as compared with the original pencil beam (PB) calculation, varied from 3% to 33%.^(2)^ Similarly, another series of 53 patients also showed PTV D95 reductions between 9.5% and 32.6% with MC calculations.^(3)^ The greatest differences were observed by Liu et al.,^(4)^ who examined 82 patients, with the variation being as high as 82.7%.

The ramifications of these large dose variations include the lack of a simple “prescribed‐to‐administered dose ratio” owing to this large uncertainty in prescribed and delivered doses. Therefore, it is imperative to fully understand the causes of these dose variations between MC and PB planning. Previous studies have shown that target dose variations between various algorithms are dependent on a multitude of factors: tumor size, tumor location, lung density, beam energy, and delivery technique.^2^, ^3^, ^5^, ^6^, ^7^, ^8^, ^9^, ^10^, ^11^ Specifically, greater target dose deficiencies are present for smaller tumors,^2^, ^3^, ^10^ peripheral tumors,^2^, ^10^ and lower lung densities.^3^, ^5^ Other factors, such as beam energy and delivery technique, were also shown to affect the target dose deficiency in more complex patterns.^6^, ^7^, ^8^

One such factor that could influence target dose differences between PB and MC planning, that has not been heretofore elucidated, is the prescription isodose line. Hence, the aim of the current study was to investigate whether this could affect the magnitude of the target dose difference between PB and MC calculations for lung SBRT. While conventional radiotherapy treatments are associated with a homogeneous target dose distribution and prescribe doses to isodose lines 90% or higher, much larger target dose heterogeneity is allowed for stereotactic radiotherapy and radiosurgery.^(12)^ Normalized prescription isodose lines 50% or even lower are not uncommon. Specifically for lung SBRT, the Radiation Therapy Oncology Group (RTOG) protocols accept a range between 60% and 90%.^13^, ^14^, ^15^ Various studies have reported prescription isodose lines at the 40%–48%,^(16)^ 50%,^(17)^ 60%,^(18)^ 65%,^19^, ^20^, ^21^, ^22^ 80%,^2^, ^3^, ^22^, ^23^, ^24^, ^25^ 85%,^(26)^ and 90%^(27)^ levels.

In the present study, we hypothesize that the normalized prescription isodose line also affects the magnitude of the target dose difference calculated between the MC and PB (conventional) algorithms. Therefore, we designed a study using both patient and virtual phantom cases, each planned at 60%, 70%, 80%, and 90% normalized prescription lines to allow for varying degrees of PTV dose heterogeneity. Because all patient‐ or tumor‐dependent confounding factors remained the same for each case, the comparison among the plans definitively illustrates the effect of varying prescription isodose lines. Our 12 patient cases were used to demonstrate the varying degrees of the isodose line effect. Using spherical tumors and uniform beam margins, the phantom cases were conducted to explain the cause of the isodose line dependence, and also to demonstrate how other factors, such as tumor size and beam energy, influence the magnitude of the isodose line effect.

To our knowledge, this is the first study examining the effect of prescription isodose lines on lung SBRT dose differences caused by calculation algorithms. Our investigation adds to the known factors contributing to the widely varying magnitude target dose differences calculated by the two algorithms, and might be useful for comparison and interpretation of treatment doses from different published studies of lung SBRT.

## II. MATERIALS AND METHODS

### A. Patient study

Under the approval of the institutional review board, 12 patients previously treated with lung SBRT at our institution were randomly selected for the study. Using the simulation CT images for each patient, treatment planning and dose investigation were conducted retrospectively. While both a 4D CT and a free‐breathing 3D CT were used to delineate the internal target volume (ITV) for clinical treatments of these patients, for the current study we followed the guidelines of RTOG 0813 and RTOG 0915,^14^, ^15^ and delineated the gross tumor volume (GTV) based only on the 3D CT. We chose to design our investigation this way because this was the standard target definition method used in most historical studies. For each patient, the GTV was delineated on the 3D CT as the gross tumor in a lung Hounsfield window. Following the previous studies, an expansion of 5 mm in the axial plane and 10 mm along the craniocaudal direction was applied to create the PTV.

Version 4.5 of the iPlan software (Brainlab AG, Feldkirchen, Germany) was used for treatment planning. For each patient, a 360° dynamic conformal arc plan with a 2 mm initial block margin was first created using a 6 MV photon beam of a TrueBeam STx linear accelerator with an HDMLC (Varian Medical Systems, Palo Alto, CA). The full arc was broken up into six subarcs so that the relative weights of the individual subarcs could be adjusted to optimize the plan quality. The MLC apertures discretized every 10° were also manually optimized to create four acceptable plans according to our clinical standards, prescribing respectively to 60%, 70%, 80%, and 90% isodose lines of the isocenter (center‐of‐mass of the PTV). For each plan, the chosen isodose line conformally covered the PTV. Therefore, after a total of 48 plans were created for the 12 patients using the iPlan PB algorithm, all plans were also recalculated using the iPlan MC algorithm^(28)^ with the original MLC apertures and monitor units. The calculated dose differences between the PB and MC dose algorithms were then compared among the four plans prescribed to the varying isodose lines (60%–90%) for each patient.

### B. Virtual phantom study

We subsequently hypothesized that the isodose line effect resulted from varied beam blocking (i.e., effective beam margins) for plans with different prescription isodose lines. In the patient plans, the MLC apertures were manually optimized for target conformity and coverage, hence nonuniform. Therefore, we designed a phantom study with uniform beam margins on simulated spherical tumors to demonstrate the causal connection between the beam margin and the isodose line effect. On the 3D CT of one patient, a virtual spherical tumor (GTV) was placed in the middle of the right lung. The density within the virtual GTV was artificially set to $1\;g/{\text{cm}}^{3}$ by overriding the density. PTV expansion and treatment planning were carried out similar to the patient study, with the exception that instead of manually optimizing the MLC apertures, a uniform block margin was selected by exploring block margin sizes from −5 mm to 5 mm, in 0.5 mm increments. Within the search range, the uniform margin size that yielded the best conformal coverage by the chosen isodose line in the axial plane was selected for each plan. PB and MC dose calculations and comparison were conducted accordingly, similar to the patient study. In addition, the virtual GTV diameters of 15 mm, 30 mm, and 50 mm were explored to investigate the influence of tumor size on the isodose line effect. Similarly, for the 30 mm diameter GTV virtual phantom case, beam energies of 6 MV, 10 MV, and 15 MV were compared to study the influence of beam energy on the isodose line effect.

### C. Dose comparison

Dose distributions and dose‐volume histograms (DVHs) were visually inspected for all plans and compared between the two dose algorithms. The relative MC vs. PB target dose differences were compared among different prescription isodose levels for each patient or phantom case.

For patient cases, the MC vs. PB differences in D95 (dose received by 95% of the volume), Dmean (mean dose), D5 (dose received by 5% of the volume) of the PTV were calculated and compared among the plans with different isodose lines. The three indices were used as quantitative representations for PTV statistical minimum (coverage) dose, mean dose, and statistical maximum dose. The PTV D98 (dose received by 98% of the volume) and D2 (dose received by 2% of the volume) were used to calculate the heterogeneity index, defined as D2/ D98, for each plan.

For virtual phantom cases, the PTV V100 (volume of the PTV that receives 100% of the prescription dose) was quantified for each plan. The MC‐to‐PB ratios of PTV V100 were compared among the plans with different isodose lines (60%, 70%, 80%, and 90%). V100, instead of D95, to assess PTV dose coverage for virtual phantom plans was chosen because the uniform block margin did not provide comparable dose coverage for the superior and inferior edges of the PTV as for the axial edges. The insufficient coverage caused a lower‐than‐prescription‐dose D95, even on the original PB plans. Two factors contributed to this directional dose coverage difference: larger GTV‐to‐PTV expansion along the superior–inferior directions (10 mm) than along the two directions in the axial plane (5 mm), and coplanar arc geometry. In patient plans, MLC apertures were manually optimized, thereby creating plans that were 3D conformal to the PTV, with D95 receiving the prescription dose. In virtual phantom plans, we wanted to maintain a uniform block margin for the purpose of investigation, so we chose to select the block margin that provided the best conformal coverage by the chosen isodose line in the axial plane, and intentionally ignored the insufficient dose coverage at the superior and inferior edges.

## III. RESULTS

A total of 48 plans were created for the 12 patient cases (4 prescription isodose lines × 12 patients), resulting in a total of 96 calculated patient plans by both the PB and MC algorithms. A total of 20 plans were created for virtual phantom cases (four prescription isodose lines × three target sizes at 6 MV + four prescription isodose lines × two other energies), resulting in 40 calculated phantom plans for comparison.

### A. Patient study

**Figure 1:**
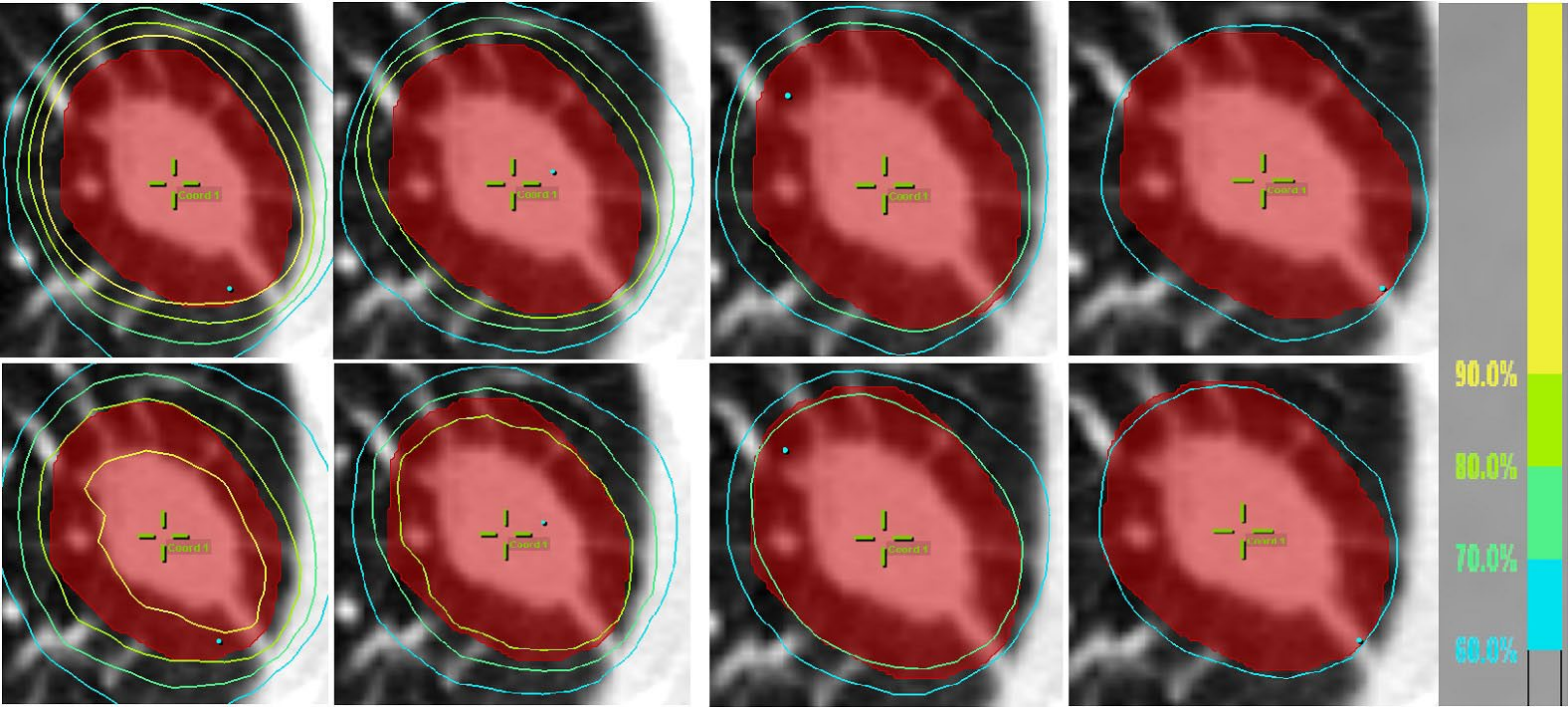
Axial dose distributions for a representative patient (Patient 5), with plans normalized to 90%, 80%, 70%, and 60% prescription lines from left to right, respectively. The red color wash illustrates the PTV. The top panels utilize PB calculations, and bottom panels the MC calculations. Isodose lines higher than the prescription line are not shown for clarity. Despite the different prescription line selections, all four original PB plans show similar coverage and conformity by their individual prescription lines. However, the coverage loss on MC‐calculated plans is apparent for the 90% plan, but becomes increasingly better for decreasing isodose line plans. The MC coverage loss on the 60% plan is almost minimal.

As expected, the MC dose calculation for all patient plans resulted in lower target dose coverage than the original PB plan, with a median (range) PTV D95 reduction of 16.0% (7.4%–28.2%). When comparing the plans with different prescription isodose lines for each patient, the following trend was observed: plans with higher prescription isodose lines were associated with greater loss of coverage by the chosen isodose line on the MC‐calculated plan (as compared with its corresponding original PB plan). Figure 1 shows a comparison of the isodose undercover‐age among the four plans with different prescription isodose lines for a representative patient. While the coverage by the chosen prescription isodose line was similar among the four original PB plans, the coverage loss on the MC‐calculated plans worsened with increasing prescription isodose lines. As is shown in Fig. 1, for example, the monotonic increase of the target coverage loss on MC plans, by isodose line, was observed for all patients.

**Figure 2:**
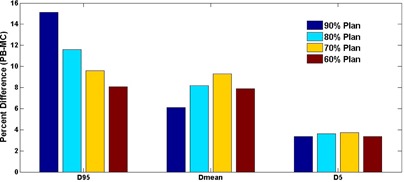
Bar graph of the PTV dose‐volume indices for a representative patient (Patient 5) showing a monotonically decreasing trend for the MC vs. PB loss in the low‐dose coverage index, D95, with decreasing prescription lines, and the lack thereof for the mean‐ and high‐dose indices (Dmean and D5).

This trend was also identified quantitatively using the studied dose‐volume indices. Figure 2 illustrates a bar plot of the dose‐volume parameters for the representative patient whose isodose distributions were shown in Fig. 1. Similar to the dose difference findings in previous studies,^(4)^ all three dose‐volume indices were lower with the MC calculation relative to the PB calculation. The reduction was the greatest for D95, less for Dmean, and the least for D5, indicating the greatest dose calculation error by PB was present at the target periphery. Comparing plans planned with different isodose lines, D95 (the statistical surrogate for the PTV minimum coverage dose) clearly showed a trend of monotonically increasing coverage loss, when calculated with MC, on plans with increasing prescription isodose lines. On the other hand, no such trend was observed for Dmean or D5 on any of the 12 patients. The results indicate that selecting different prescription isodose lines mostly affects PTV dose loss at the peripheral low‐dose region, with the clear trend that a lower prescription isodose line alleviates the coverage dose loss compared with a higher line, but it does not affect the dose loss at the hot spots within the target (the central high‐dose region).

**Figure 3:**
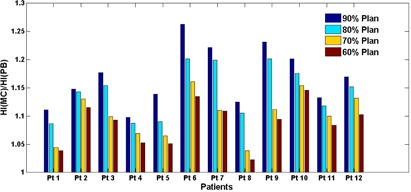
Bar graph showing the MC‐to‐PB ratios of the heterogeneity index compared among the four different prescription isodose line plans for all 12 patients. The same trend can be observed that the ratio is higher for plans with higher prescription lines. In other words, while MC calculation always leads to a higher plan heterogeneity index than its original PB plan (the ratio is always >1), the relative increase is greater for plans with higher prescription lines than those with lower prescription lines.

As expected, the PTV heterogeneity was always greater on MC‐calculated plans compared with the PB originals. However, the magnitude of the heterogeneity difference between MC and PB calculations was also found to depend on the isodose line. As a result of the coverage dose loss being dependent on the prescription isodose line, though decreasing prescription isodose lines led to larger target dose heterogeneity, the relative increase of the heterogeneity on MC‐calculated plans was actually smaller. Figure 3 depicts a bar plot of the MC‐to‐PB ratio of heterogeneity indices versus the prescription isodose line for all patients. This trend was clear on each of the 12 studied patients.

### B. Virtual phantom study

Table 1 lists the optimized uniform block margin sizes for all phantom plans using the 6 MV beams. An inspection of the optimal margin sizes first confirmed the correlation between block margin size and prescription isodose line: on a given target, the optimal uniform block margin was always smaller for a plan prescribed to a lower isodose line than that to a higher line. Comparing targets of varying sizes, a larger target required a smaller optimal block margin.

**Figure 4:**
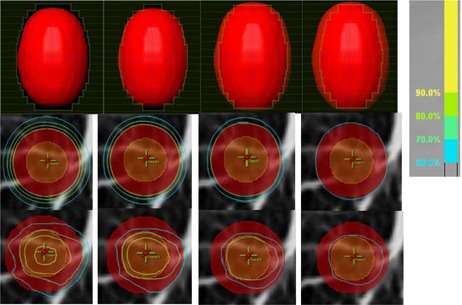
Beam's‐eye views (top panels) and axial view dose distributions (middle panels for PB calculation and bottom panels for MC calculation) for the 15 mm diameter spherical GTV virtual phantom case planned with 6 MV energy, with 90%, 80%, 70%, and 60% prescription lines from left to right, respectively. The PTV is in red color wash and the GTV is in orange color wash. As shown in the beam's‐eye views, a uniform block margin of 0.5 mm, −0.5 mm,−1.5 mm, and −2.5 mm was used for the four plans from 90% to 60% isodose lines, respectively, resulting in similar axial coverage on the PB plans. Similar to the patient‐case findings, the MC vs. PB coverage loss is apparent, with the loss being more pronounced with higher prescription line (corresponding to a larger block margin) than for a lower prescription line (corresponding to a smaller block margin).

With the phantom cases, the same trend of larger MC vs. PB target dose losses with higher prescription isodose line plans was also observed. Figure 4 shows an example phantom case with the beam's‐eye views and axial isodose distributions for the plans on the 15 mm diameter spherical GTV virtual phantom with 6 MV photons. The PB plans with varying prescription isodose lines showed similarly conformal coverage in the axial view. In contrast, the MC‐recalculated plans afforded poorer target coverage when the prescription isodose line was higher.

Table 2 tabulates the MC‐to‐PB PTV V100 ratios for all 6 MV phantom plans prescribed to the varying isodose lines for different target sizes. The ratios were always <1, because PB overestimated the target dose compared with MC. Comparing the ratios for plans with different isodose lines on a target of given size, the ratio monotonically decreased with a higher isodose line, indicating a larger MC vs. PB coverage loss for a higher isodose line (consistent with the aforementioned patient data). This result quantitatively confirmed the observed isodose line trend, in direct connection with varying sizes of the uniform block margin. In addition, the target size was shown to affect the magnitude of the isodose line dependence; this magnitude was greater for a smaller target size.

**Table 1:** Optimal uniform block margin sizes used for virtual phantom plans for spherical GTVs of 15 mm, 30 mm, and 50 mm diameters, planned individually with 90%, 80%, 70%, and 60% prescription isodose lines and with 6 MV photon beams, as based on PB calculation

*Block Margin (mm)*	Rx Line=90%	Rx Line=80%	Rx Line=70%	Rx Line=60%
$D_{\text{GTV}}=15\;\text{mm}$	0.5	−0.5	−1.5	−2.5
$D_{\text{GTV}}=30\;\text{mm}$	0	−0.5	−1.5	−3.5
$D_{\text{GTV}}=50\;\text{mm}$	−0.5	−1.5	−3	−5

Table 3 denotes the MC‐to‐PB PTV V100 ratios for the 30 mm diameter GTV phantom using 6 MV, 10 MV, and 15 MV photon plans. The isodose line trend was observed for all energies, and the magnitude of its effect was found to be greater with a higher beam energy.

**Table 2:** MC‐to‐PB ratios of PTV V100 for virtual phantom cases with spherical GTVs of 15 mm, 30 mm, and 50 mm diameters, planned individually with 90%, 80%, 70%, and 60% prescription isodose lines and with 6 MV photon beams, as based on PB calculation

*V100(MC)/V100(PB)*	Rx Line=90%	Rx Line=80%	Rx Line=70%	Rx Line=60%
$D_{\text{GTV}}=15\;\text{mm}$	0.01	0.09	0.17	0.27
$D_{\text{GTV}}=30\;\text{mm}$	0.23	0.31	0.47	0.57
$D_{\text{GTV}}=50\;\text{mm}$	0.48	0.54	0.70	0.78

**Table 3:** MC‐to‐PB ratios of PTV V100 for virtual phantom cases with a spherical GTV of 30 mm diameter, planned individually with 90%, 80%, 70%, and 60% prescription isodose lines and with 6 MV, 10 MV, and 15 MV photon beams, as based on PB calculation

*V100(MC)/V100(PB)*	Rx Line=90%	Rx Line=80%	Rx Line=70%	Rx Line=60%
Energy=6 MV	0.23	0.31	0.47	0.57
Energy=10 MV	0.13	0.27	0.40	0.49
Energy=15 MV	0.07	0.23	0.35	0.45

## IV. DISCUSSION

Unlike previous studies that used “interpatient” comparisons to study the effect of factors such as tumor size and location,^2^, ^10^ our study used “intrapatient” comparisons to study the effect of normalized prescription isodose lines on dose variations between PB and MC algorithms. This study design is similar to a previously published phantom study by Aarup et al.,^(5)^ who investigated the effect of various lung densities on target coverage differential. With these approaches, all other confounding factors were “controlled,” and therefore a large number of cases were not required for statistical power in the comparison. The comparison in each case was therefore definitive, purely illustrating the effect of the studied variable — the prescription isodose line in our case. In fact, the same trend was unequivocally shown for all 12 patients in our study, and the dependence of the target dose variations on the prescription isodose line was monotonic in every case.

A clear trend dependent on the prescription isodose line was only observed for target minimum dose indices such as PTV D95 and D98, but not for the mean/maximum dose indices such as Dmean, D5, or D2. In other words, the isodose line effect showed a trend for dose errors only to the peripheral target. While MC also calculated lower central‐target doses than PB, the difference did not correlate with the prescription isodose line.

**Figure 5:**
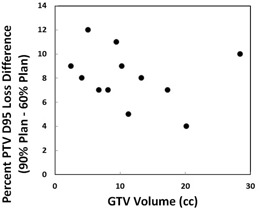
Isodose line dependent percent PTV D95 loss (90% plan vs. 60% plan) vs. GTV volume for all 12 patients. No apparent correlation can be identified due to other confounding factors such as tumor location and lung density.

For different patients, the magnitude of the observed isodose line effect was influenced by all other confounding factors such as tumor size, tumor location, lung density, and beam energy. Therefore, to quantify the isodose line effect based on the value of a single confounding factor would not be practical. For example, in Fig. 5 we plotted the isodose line‐dependent percent PTV D95 loss (90% plan vs. 60% plan) against the GTV volume for all 12 patients. No apparent correlation was identified due to other confounding factors such as tumor location and lung density. In contrast, in our phantom study where these confounding factors were more easily controlled, the quantitative results were achievable, as reported in Tables 2 and 3. The isodose line‐associated coverage loss differences were quantified at different GTV sizes and with different beam energies by the phantom study. For the 12 patient cases we investigated, the greatest isodose line‐associated difference was seen for one patient at 18% PTV D95 loss for the 90% prescription line plan, and 6% loss for the 60% prescription line plan, rendering a 12% difference between the two extremes. The smallest difference was seen for another patient at 12% and 8% respectively, amounting to a 4% overall difference.

It stands to reason that the major difference among plans with different isodose lines resulted from the varying effective beam margins. A 60% isodose line plan used a much smaller effective beam margin than a 90% isodose line plan for the same patient. Therefore, varying amounts of low‐density lung tissue in the beam margin directly influenced the dose calculation accuracy at the interface (i.e., at the periphery of the target). To more clearly illustrate this effect, the phantom study was designed using stylized (spherical) tumors and a uniform block margin for each plan. Table 1 clearly shows that the required block margin size monotonically increased for plans with increasing prescription isodose lines. The correlation between the margin size and the prescription line, hence the MC vs. PB target dose loss, is also illustrated for the example case in Fig. 4. Additionally, Table 2 demonstrates that an increase in the MC‐calculated dose coverage loss relative to PB was caused by an increase in margin size, and hence from the PB calculation error in the region of the exposed low‐density tissue.

One limitation of our study was that we used PTV as the target surrogate for all analyses. Our choice of PTV endpoints such as PTV D95 was because this has been the standard clinical practice. PTV is the prescription surrogate that radiation oncologists are accustomed to using and is how they perceive target dose. PTV D95 is used for prescription by the lung SBRT protocols^14^, ^15^ and has been heretofore used by most study reports. On the other hand, while still the current standard practice, the appropriateness of PTV‐based prescription in lung SBRT planned with accurate heterogeneity correction has been called into question.^3^, ^22^, ^29^, ^30^ As pointed out by Lacornerie et al.,^(29)^ in lung SBRT PTV D95 will likely be calculated in a region where there is no electronic equilibrium. The conventional prescription to PTV D95 will therefore depend mainly on lung density and does not predict dose to GTV, the real target, which is dense, because of the rebuildup effect. Furthermore, there are generally fewer histories in a low‐density region, so the MC calculation uncertainty is higher. Therefore, PTV, which was a fictitious volume initially created to ensure that the absorbed dose to the true target equals the prescription dose, taking into account positioning uncertainties, may no longer be a suitable surrogate for dose prescription in MC‐based lung SBRT. Instead, fluence optimization based on the PTV and dose prescription based on the GTV might be more appropriate. However, since PTV‐based dose was reported in the vast majority of literatures and PTV D95‐based prescription is still the current standard clinical practice,^14^, ^15^ we conducted our study using PTV‐based target dose end points. This choice was also consistent with our purpose of investigating the isodose line effect to understand if it in addition to the known factors contributed to the large variation of previously‐reported PTV D95 differences for individual patients. We would like to note, though, with GTV‐based end points, the isodose line effect might be different from what was found in our study.

Taken together, to our knowledge, this is the first report on the dependence of MC‐calculated target dose loss (as compared with less accurate algorithms such as PB) on the normalized prescription isodose line. This information is useful to re‐examine the algorithm‐associated dose variations that are extensively reported in the literature, and in the future application of dose correction to historical data wherein a wide range of prescription isodose lines were utilized. New radiobiological models based on accurate dose calculations and known clinical outcomes could then guide clinical implementation of MC‐based treatment planning for lung SBRT.

## V. CONCLUSION

Variations in the MC vs. PB PTV dose loss based on selected prescription isodose lines were identified for lung SBRT. Dose losses are more pronounced with higher prescription isodose lines. The trend is caused by the varying margin sizes used for different prescription levels, and shows a greater magnitude for smaller tumors and higher beam energies. This newly identified dependence should be taken into consideration when re‐evaluating historical cases calculated by PB‐type algorithms, especially cases that utilized different prescription isodose lines, in order to correlate clinical outcomes with realistic target doses calculated by the more accurate MC‐type dose algorithms.

## COPYRIGHT

This work is licensed under a Creative Commons Attribution 3.0 Unported License.

## Supporting information

Supplementary Material

Supplementary Material

Supplementary Material
