# Supplementary figures and images for: Effect of the normalized prescription isodose line on the magnitude of Monte Carlo vs. pencil beam target dose differences for lung stereotactic body radiotherapy
Source: J Appl Clin Med Phys. 2016 Jul 8;17(4):48–58. doi: 10.1120/jacmp.v17i4.5965 (PMC5690053; doi:10.1120/jacmp.v17i4.5965)

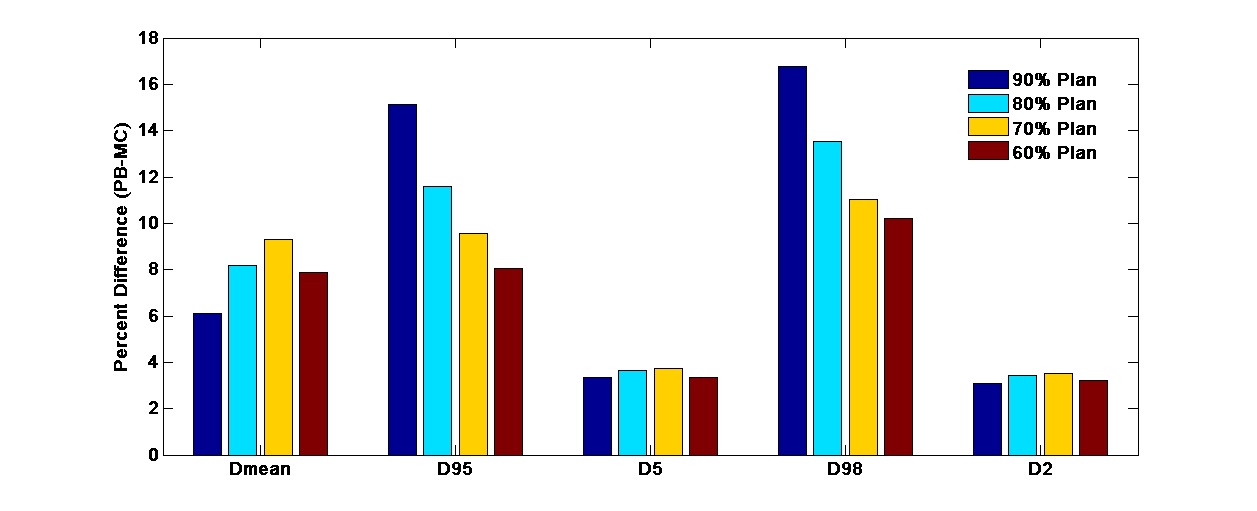

Supplement: Supplementary file 1 — Supplementary Material [file ACM2-17-048-s001.jpg]

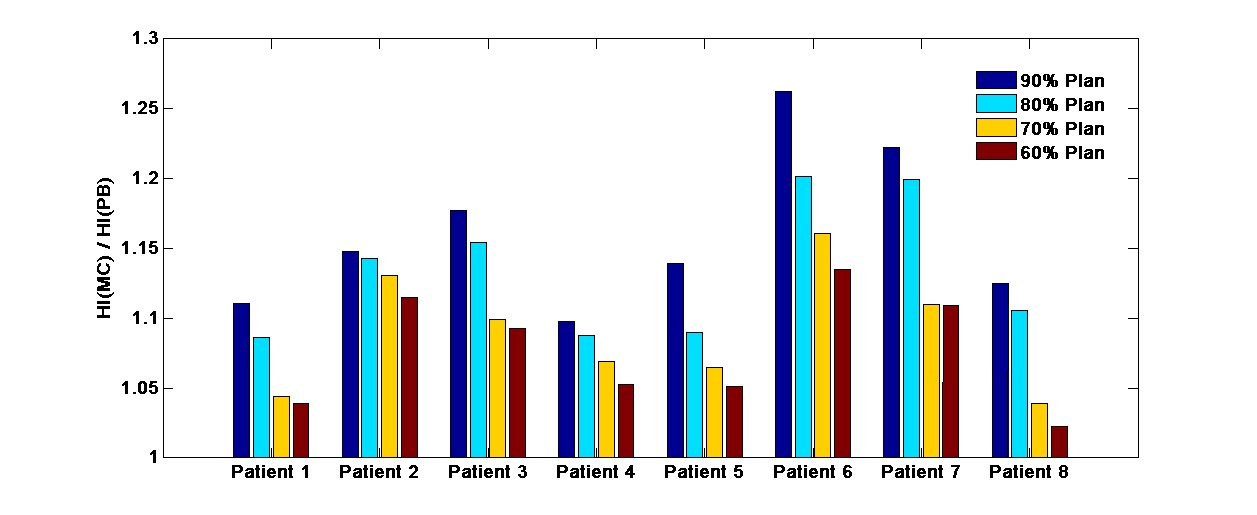

Supplement: Supplementary file 2 — Supplementary Material [file ACM2-17-048-s002.jpg]

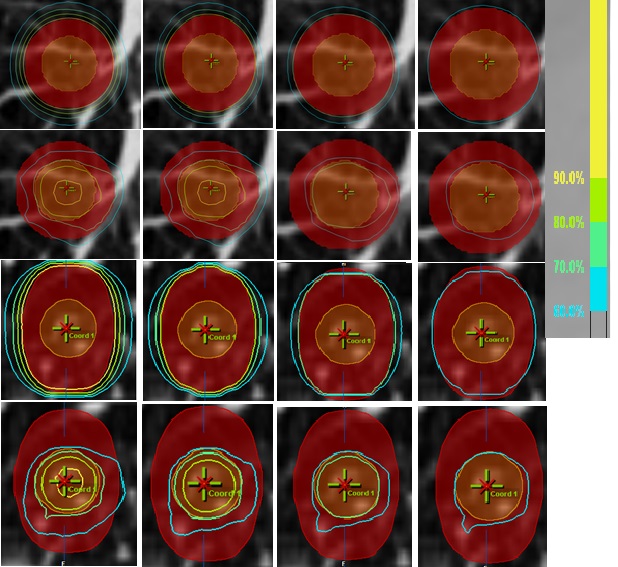

Supplement: Supplementary file 3 — Supplementary Material [file ACM2-17-048-s003.jpg]
